# Selected Derivatives of Erythromycin B-*In Silico* and Anti-Malarial Studies

**DOI:** 10.3390/ma14226980

**Published:** 2021-11-18

**Authors:** Pranab K. Bhadra, Rachael N. Magwaza, Niroshini Nirmalan, Sally Freeman, Jill Barber, Biljana Arsic

**Affiliations:** 1 Division of Pharmacy and Optometry, School of Health Sciences, University of Manchester, Oxford Road, Manchester M13 9PT, UK; bhadra.pranab@gmail.com (P.K.B.); rachael.magwaza@manchester.ac.uk (R.N.M.); Sally.Freeman@manchester.ac.uk (S.F.); Jill.Barber@manchester.ac.uk (J.B.); 2 School of Science, Engineering & Environment, University of Salford, Manchester M5 4WT, UK; n.j.nirmalan@salford.ac.uk; 3 Department of Chemistry, Faculty of Sciences and Mathematics, University of Nis, Visegradska 33, 18000 Nis, Serbia

**Keywords:** erythromycin B derivatives, anti-malarial activity, conformational search, molecular docking, interactions

## Abstract

Erythromycin A is an established anti-bacterial agent against Gram-positive bacteria, but it is unstable to acid. This led to an evaluation of erythromycin B and its derivatives because these have improved acid stability. These compounds were investigated for their anti-malarial activities, by their *in silico* molecular docking into segments of the exit tunnel of the apicoplast ribosome from *Plasmodium falciparum*. This is believed to be the target of the erythromycin A derivative, azithromycin, which has mild anti-malarial activity. The erythromycin B derivatives were evaluated on the multi-drug (chloroquine, pyrimethamine, and sulfadoxine)-resistant strain K1 of *P. falciparum* for asexual growth inhibition on asynchronous culture. The erythromycin B derivatives were identified as active in vitro inhibitors of asexual growth of *P. falciparum* with low micro-molar IC_50_ values after a 72 h cycle. 5-Desosaminyl erythronolide B ethyl succinate showed low IC_50_ of 68.6 µM, *d*-erythromycin B 86.8 µM, and erythromycin B 9-oxime 146.0 µM on the multi-drug-resistant K1 of *P. falciparum*. Based on the molecular docking, it seems that a small number of favourable interactions or the presence of unfavourable interactions of investigated derivatives of erythromycin B with *in silico* constructed segment from the exit tunnel from the apicoplast of *P*. *falciparum* is the reason for their weak in vitro anti-malarial activities.

## 1. Introduction

Malaria remains a huge health problem worldwide with 229 million cases and 409,000 registered deaths reported in 2019 [1]. Due to the growing resistance from Southeast Asia to Africa to almost all anti-malarial drugs, it is clear that new therapeutic strategies are essential. Compounds like reversal agents (chemosentisizers, modulating agents) can restore the sensitivity in resistant cells [2,3]. Erythromycin A was first discovered as a reversal agent in multidrug-resistant cancer [4,5]. Antibiotics were found to possess anti-plasmodial activity as early as 1940, but they were shown to be less effective and slow acting compared to chloroquine or quinine [3]. Erythromycin A possesses in vitro and in vivo anti-malarial activity [6]. Its anti-malarial action is improved in combination with chloroquine; therefore, its modulating effect is important in chloroquine resistance [3]. Anti-malarial activity has been described for other antibiotics such as co-trimoxazole, quinolones, tigecycline, mirincamycin, ketolides, fusidic acid, and thiopeptides [7]. Based on studies in Africa, co-trimoxazole can be used as an alternative in malaria prophylaxis, for children and adults, HIV (human immunodeficiency viruses) positive or negative patients and pregnant women [8,9,10,11]. Tigecycline must be administered intravenously; therefore, its use is reserved for patients with severe and complicated malaria, with delayed action similar to doxycycline [7].

The search for more active anti-malarial macrolides than the commercially available anti-malarial drugs has continued. A research group at GSK (GlaxoSmithKline) in Zagreb working on macrolides has published a series of ureas and thioureas of 15-membered azalides with the ureas containing a naphthyl moiety being the most active and 9a-*N* substituted 15-membered azalides with amide and amine functional groups with improved in vivo efficacy [12,13].

Azithromycin was shown to be a slow anti-malarial targeting parasite apicoplast (an organelle with four membranes containing 35 kb circular DNA (deoxyribonucleic acid) with replication, RNA (ribonucleic acid) transcription, and RNA-protein translation allowed [14]), giving IC_50_ in the nanomolar range [15], but better in combination with existing anti-malarial drugs [16].

**Figure 1:**
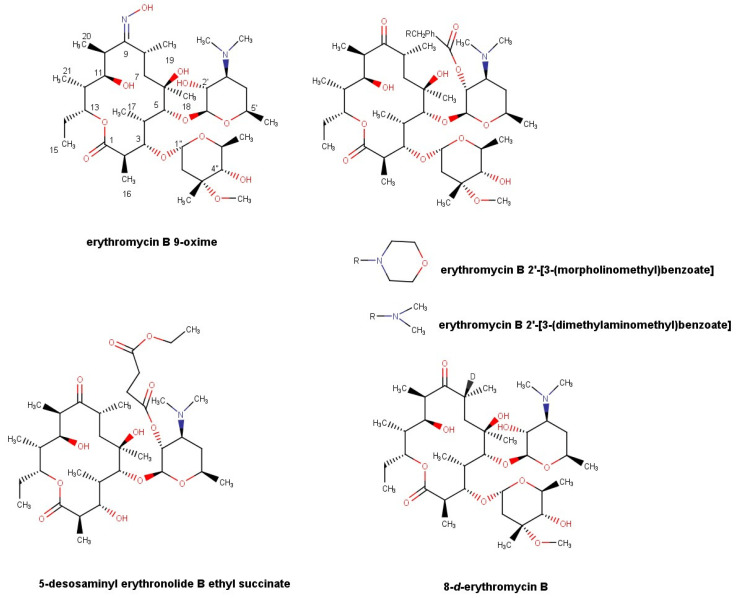
Investigated derivatives of erythromycin B against *P. falciparum* K1 strain.

Therefore, developing a more effective and safer anti-malarial from the class of macrolides is the aim of this research. Here, the in vitro anti-malarial activity of synthesised derivatives of erythromycin B (erythromycin B 9-oxime, 5-desosaminyl erythronolide B ethyl succinate, erythromycin B 2′-[3-(morpholinomethyl)benzoate], erythromycin B 2′-[3-(dimethylaminomethyl)benzoate], and 8-*d*-erythromycin B) (Figure 1), an acid-stable derivative of erythromycin A [17] have been investigated, including the use of molecular docking to give insight regarding the mechanism of action.

## 2. Materials and Methods

### 2.1. Instrumentation

#### 2.1.1. NMR (Nuclear Magnetic Resonance) Spectrometers

##### Bruker Avance 300 Spectrometer

A Bruker Avance 300 spectrometer (Bruker, Billerica, MA, USA) was operated with an Oxford Instruments magnet (Billerica, MA, USA, magnetic field—7.04 Tesla). In this field, the ^1^H resonated at 300 MHz and the ^13^C at 75.47 MHz. A 5 mm QNP (Quattro Nucleus Probe–^1^H, ^13^C, ^19^F, ^31^P) probe with Z gradients was used.

##### Varian Unity 500 Spectrometer

A Varian Unity 500 NMR spectrometer (Varian Medical Systems, Palo Alto, CA, USA) was operated with an Oxford Instruments magnet, magnetic field—11.74 Tesla. In this field, the ^1^H resonated at 500 MHz. A 5 mm PFG (Pulse Field Gradient) probe was used. The temperature was controlled using the Varian VT unit with a precision of ±0.1 °C.

##### Bruker AMX 500 Spectrometer

A Bruker AMX 500 (Bruker, Billerica, MA, USA) was operated with an Oxford Instruments magnet, magnetic field—11.74 Tesla. Either a TXI-Z (triple nucleus inverse probe with Z gradients) or a BBI (broad band inverse probe) was used.

The variable temperature unit was used as required. NMR tubes, 507-PP (Wilmad, NJ, USA) were used in all experiments, unless otherwise stated. The spectrometers were used to acquire one-dimensional (^1^H, ^13^C, DEPT (Distortionless Enhancement by Polarisation Transfer)-45, DEPT-90 and DEPT-135) and two-dimensional (DQF-COSY, TOCSY, DOSY, HMQC, and HMBC) spectra. The spectra were either acquired in deuterated chloroform (CDCl_3_) referenced to tetramethylsilane (TMS) (Lancaster, UK) as an internal standard or in deuterated buffer (apparent pH 2.0, 6.0, and 7.0) referenced to 3-(trimethylsilyl)-2,2,3,3-tetradeuterio-propionic acid sodium salt (TSP) (Aldrich, Gillingham UK).

#### 2.1.2. Mass Spectrometer

Electrospray ionisation-mass spectra (ESI-MS) were acquired on a Micromass Platform mass spectrometer (Waters Corporation, Milford, MA, USA), and the data were analysed using the program PLATFORM with a Masslynx data system. The sample (10 μL) was injected using a Hewlett Packard auto-sampler, and the machine was operated at a cone value of 30 eV, at 80 °C. For identification purposes, all samples (0.2 mg) were prepared in acetonitrile (1 mL). For degradation studies, a deuterated Britton–Robinson buffer was used unless otherwise stated. Water was used as the solvent for running the samples unless otherwise stated.

#### 2.1.3. pH Meter

All pH measurements were carried out using a Mettler Delta 320 (Mettler Toledo, Greifensee, Switzerland), equipped with a combination electrode (Gelpas, BDH, Poole, UK). The pH of the deuterated buffers was measured on the Hanna pH 213 (Hanna Instruments Inc., Woonsocket, RI, USA), equipped with a combination electrode (Gelpas, BDH, Poole, UK) and a temperature probe. Buffer reference standards (4.00 ± 0.01, 7.00 ± 0.01, and 10.00 ± 0.01 at 25 °C) (Sigma-Aldrich, Saint Louis, MO, USA) were used to calibrate the pH meters by a two-point calibration method. All pH measurements were at room temperature and are reported uncorrected.

#### 2.1.4. Melting Point Apparatus

All melting points were determined using a Bibby SMP-10 (Stuart Scientific, Staffordshire, UK) melting point apparatus and are reported uncorrected. The samples were placed in a capillary tube and inserted in the melting point apparatus and were heated at a rate of 1 °C/min until the compound melted.

#### 2.1.5. Thin Layer Chromatography (TLC)

All the TLC experiments were carried on already-prepared aluminium-backed silica gel 60 F_254_ (Merck, Darmstadt, Germany) plates without any prior activation. Spots were applied using a 5 μL capillary, and EtOAc-CH_3_OH-25% NH_3_ (85:10:5) was used as the mobile phase [18]. The plates were dried in the oven for 1 min and then were sprayed with *p*-anisaldehyde: sulphuric acid: ethanol mixture (1:1:9). The plates were dried in the oven set at 110 °C for 5 min, and the R_f_ value for each of the compounds was determined.

### 2.2. Two-Dimensional NMR Spectroscopy

#### 2.2.1. TOCSY and DQF-COSY

For structural determination, both the TOCSY (Total Correlated Spectroscopy) and the DQF-COSY (Double Quantum Filtered Correlated Spectroscopy) spectra were obtained at ambient temperature on the Varian Unity 500 spectrometer. In the TOCSY experiment, cross-peaks were found for all the protons in the same spin system. The COSY spectrum indicated all the spin–spin coupled protons. For both the experiments, 2048 × 2048 complex points were acquired. The spectra were processed using negative line broadening and Gaussian multiplication for resolution enhancement.

#### 2.2.2. HMQC and HMBC

HMBC (Heteronuclear Multiple Bond Connectivity) and HMQC (Heteronuclear Multiple Quantum Coherence) spectra were acquired on the Varian Unity 500 spectrometer. An HMBC spectrum was acquired by using a long-range ^1^H-^13^C COSY pulse sequence to give a two-dimensional spectrum with ^13^C chemical shifts on one axis and ^1^H chemical shifts on the other. An HMQC indicated single-bond correlation between ^13^C and the proton to which, it was directly attached.

#### 2.2.3. DOSY

Erythromycin B 2′-ethyl succinate was dissolved in deuterated Britton–Robinson buffer at an apparent pH 2.0 to give concentration of 4 mM and was degraded over two hours. The degraded samples were cooled to ambient temperature and a high resolution DOSY (Diffusion-Ordered Spectroscopy) spectrum was acquired using a ONESHOT DOSY pulse sequence. Twelve spectra were acquired, with gradient pulses of 2.9 ms. The FIDDLE (free induction decay deconvolution for line shape enhancement) algorithm [19] was used to correct line shapes using TSP as a reference standard. DOSY spectra were constructed after baseline correction by taking the first echo spectrum and distributing the intensities of the individual signals in the second dimension according to their respective diffusion coefficient [20,21].

### 2.3. Synthesis of Erythromycin B Derivatives

Erythromycin B was a gift from Dr. Warren Mann, Abbott International Division, which was used without further purification. Acetone (GPR grade, BDH, Poole, UK) dried over anhydrous sodium sulphate (Na_2_SO_4_) was used as a solvent in most syntheses, unless stated otherwise. The water work-up refers to washing the organic layer with water (2 × 50 mL) and 50 mL brine (saturated sodium chloride solution).

#### 2.3.1. Synthesis of Erythromycin B Oxime

##### The First Method

This method was adapted from Gasc et al. (1991) [22]. Erythromycin B (5.0 g) was dissolved in 50 mL methanol (GPR grade, BDH, UK, previously dried over sodium sulphate) followed by addition of 2.46 mL triethylamine and 2.46 g hydroxylamine hydrochloride (Sigma-Aldrich, Burlington, MA, USA). The mixture was refluxed for 24 h. After complete dissolution, the progressive crystallisation of erythromycin B oxime hydrochloride was observed. The reaction mixture was cooled to 2 °C for 1 h. The crystals were separated and washed three times with 2.5 mL cooled methanol. To the suspension of erythromycin oxime hydrochloride in 15 mL methanol (20 °C), 3.26 mL of ammonium hydroxide solution (20%) was added. After stirring for 30 min, 3.26 mL of water was added to the solution to give 1.8 g of erythromycin B oxime. Yield 36.0%, m.p. 170–174 °C, ^1^H NMR (CDCl_3_, 300 MHz): δ 0.87 (CH_3_-21, d, *J* = 7.1 Hz), δ 0.87 (CH_3_-15, t, *J* = 7.3 Hz), δ 1.05 (CH_3_-20, d, *J* = 6.8 Hz), δ 1.12 (CH_3_-17, d, *J* = 7.5 Hz), δ 1.13 (CH_3_-19, d, *J* = 7.1 Hz), δ 1.18 (CH_3_-16, d, *J* = 7.3 Hz), δ 1.21 (CH_3_-6′, d, *J* = 6.0 Hz), δ 1.21 (CH_3_-7″, s), δ 1.29 (CH_3_-6″, d, *J* = 6.2 Hz), δ 1.5 (CH_3_-18, s), δ 2.28 (N(CH_3_)_2_-7′,8′, s), δ 2.89 (CH-2, dq, *J* = 9.0, 7.1 Hz), δ 3.21 (CH-2′, dd, *J* = 10.3, 7.5 Hz), δ 3.31 (OCH_3_-8″, s), δ 3.49 (CH-5′, m), δ 3.6 (CH-5, d, *J* = 7.5 Hz), δ 3.63 (CH-11, d, *J* = 10.0 Hz), δ 4.0 (CH-5″, dq, *J* = 9.5, 6.2 Hz), δ 4.06 (CH-3, dd, *J* = 9.0, 1.5 Hz), δ 4.43 (CH-1′, d, *J* = 7.3 Hz), δ 4.93 (CH-1″, d, *J* = 4.7 Hz), δ 5.42 (CH-13, dd, *J* = 9.6, 4.6 Hz); *m/z* 733 [M + H]^+^; R_f_ 0.36 (CH_2_Cl_2_-CH_3_OH-25% NH_3_, 90:9:1.5) [18]; Anal. Calcd for C_37_H_68_N_2_O_12_ (%): C 60.62, H 9.37, N 3.82. Found (%): C 60.42, H 9.29, N 3.71.

##### The Second Method

This method was adapted from Watanabe et al. (1993) [23]. Erythromycin B (5.0 g), 2.49 g anhydrous sodium acetate (Fisher Scientific, Loughborough, UK) and 1.75 g hydroxylamine hydrochloride (Sigma-Aldrich, Burlington, MA, USA) were added to 20 mL methanol, and the suspension was stirred at room temperature for 6 days and then refluxed for 30 min. The reaction mixture was concentrated *in vacuo* to about half the original volume and poured into 10 mL water. The resulting crystals were filtered, triturated successively with water, saturated NaHCO_3_ solution and water, and then filtered. Crystallisation from dichloromethane-*n*-hexane gave 3.46 g of erythromycin B oxime. Yield 69.2%, m.p. 172–174 °C, ^1^H NMR (CDCl_3_, 500 MHz): δ 0.89 (CH_3_-21, d, *J* = 7.1 Hz), δ 0.88 (CH_3_-15, t, *J* = 7.3 Hz), δ 1.03 (CH_3_-20, d, *J* = 6.8 Hz), δ 1.03 (CH_3_-16, d, *J* = 7.3 Hz), δ 1.12 (CH_3_-17, d, *J* = 7.5 Hz), δ 1.18 (CH_3_-19, d, *J* = 7.1 Hz), δ 1.25 (CH_3_-6′, d, *J* = 6.0 Hz), δ 1.25 (CH_3_-7″, s), δ 1.32 (CH_3_-6″, d, *J* = 6.2 Hz), δ 1.51 (CH_3_-18, s), δ 2.27 (N(CH_3_)_2_-7′,8′, s), δ 2.89 (CH-2, dq, *J* = 9.0, 7.1 Hz), δ 3.0 (CH-10, dq, *J* = 6.8, 1.1 Hz), δ 3.23 (CH-2′, dd, *J* = 10.3, 7.5 Hz), δ 3.33 (OCH_3_-8″, s), δ 3.49 (CH-5′, m), δ 3.58 (CH-5, d, *J* = 7.5 Hz), δ 3.59 (CH-11, bd, *J* = 10.0 Hz), δ 4.0 (CH-5″, dq, *J* = 9.5, 6.2 Hz), δ 4.06 (CH-3, dd, *J* = 9.0, 1.5 Hz), δ 4.42 (CH-1′, d, *J* = 7.3 Hz), δ 4.92 (CH-1″, d, *J* = 4.7 Hz), δ 5.42 (CH-13, dd, *J* = 9.6, 4.6 Hz); *m/z* 733 [M+H]^+^; R_f_ 0.23 (CH_2_Cl_2_-CH_3_OH-25% NH_3_, 90:9:1.5); Anal. Calcd for C_37_H_68_N_2_O_12_ (%): C 60.62, H 9.37, N 3.82. Found (%): C 60.51, H 9.63, N 3.66.

#### 2.3.2. Synthesis of Erythromycin B 2′-(3-chloromethylbenzoate) (EBCMB)

EBCMB acts as a starting material for all the erythromycin 2′-(*N*-substituted 3- or 4- aminomethylbenzoate) esters. It was prepared by adapting the procedure used by Jensen et al. (1990) [24]. 3-Chloromethylbenzoyl chloride (1.25 mL, Sigma-Aldrich, Burlington, MA, USA) in 5 mL acetone was added dropwise to a mixture of erythromycin B (5 g) and 1 g NaHCO_3_ in 200 mL acetone. The resulting solution was stirred at room temperature overnight. The residue was stirred with 100 mL phosphate buffer (pH 6.5) for 15 min followed by a water work-up. The organic layer was dried *in vacuo*, and the crystalline product was recrystallised from ethyl acetate. Yield 89.4%, m.p. 192–194 °C, ^1^H NMR (CDCl_3_, 500 MHz): δ 0.72 (CH_3_-17, d, *J* = 7.1 Hz), δ 0.76 (CH_3_-21, d, *J* = 7.5 Hz), δ 0.82 (CH_3_-15, t, *J* = 7.4 Hz), δ 0.96 (CH_3_-20, d, *J* = 6.8 Hz), δ 1.12 (CH_3_-19, d, J = 7.1 Hz), δ 1.16 (CH_3_-16, d, *J* = 7.1 Hz), δ 1.26 (CH_3_-6′, d, *J* = 6.2 Hz), δ 1.27 (CH_3_-7″, s), δ 1.29 (CH_3_-6″, d, *J* = 6.2 Hz), δ 1.46 (CH_3_-18, s), δ 2.25 (N(CH_3_)_2_-7′,8′, s), δ 2.36 (CH-2″, d, *J* = 15.2 Hz), δ 3.41 (OCH_3_-8″, s), δ 3.55 (CH-5, d, 7.5 Hz), δ 3.69 (CH-11, d, *J =* 9.8 Hz), δ 4.0 (CH-5″, obs), δ 4.61 (CH_2_-16′, s), δ 4.70 (CH-1′, d, *J =* 7.5 Hz), δ 4.86 (CH-1″, d, *J =* 4.5 Hz), δ 5.02 (CH-2′, dd, *J =* 10.5, 7.5 Hz), δ 5.28 (CH-13, dd, *J =* 9.4, 4.8 Hz), δ 7.42 (CH-12′, dd, *J =* 7.7 Hz), δ 7.57 (CH-13′, d, *J =* 7.7 Hz), δ 7.96 (CH-11′, d, *J =* 7.7 Hz), δ 8.05 (CH-15′, s); *m/z* 870 [M + H]^+^; R_f_ 0.62 (EtOAc-CH_3_OH-25% NH_3_, 85:10:5); Anal. Calcd for C_45_H_72_NO_13_Cl (%): C 62.08, H 8.35, N 1.60. Found (%): C 62.05, H 8.40, N 1.59.

#### 2.3.3. Synthesis of Erythromycin B 2′-[3-(morpholinomethyl)benzoate] (EBMMB)

A mixture of erythromycin B 2′-(3-chloromethylbenzoate) (1 g), morpholine (0.2 mL) and sodium iodide (20 mg) in 75 mL acetone was refluxed for 20 h. Following the water work-up the organic layer was reduced to dryness *in vacuo*. The colourless crystals of EBMMB were recrystallised from ethyl acetate. Yield 83%, m.p. 126–128 °C, ^1^H NMR (CDCl_3_, 500 MHz): δ 0.7 (CH_3_-17, d, *J* = 7.1 Hz), δ 0.76 (CH_3_-21, d, *J* = 7.5 Hz), δ 0.84 (CH_3_-15, t, *J* = 7.4 Hz), δ 0.96 (CH_3_-20, d, *J* = 6.8 Hz), δ 1.13 (CH_3_-19, d, *J* = 7.3 Hz), δ 1.17 (CH_3_-16, d, *J* = 6.8 Hz), δ 1.26 (CH_3_-6′, d, *J* = 6.2 Hz), δ 1.28 (CH_3_-7″, s), δ 1.30 (CH_3_-6″, d, *J* = 6.2 Hz), δ 1.46 (CH_3_-18, s), δ 2.26 (N(CH_3_)_2_-7′,8′, s), δ 3.42 (OCH_3_-8″, s), δ 3.49 (CH-5′, m), δ 3.58 (CH-5, d, *J* = 7.5 Hz), δ 3.69 (CH-11, d, *J* = 10.0 Hz), δ 4.0 (CH-5″, dq, *J* = 9.4, 6.2 Hz), δ 4.0 (CH-3, dd, *J* = 9.0, 1.5 Hz), δ 4.70 (CH-1′, d, *J* = 7.5 Hz), δ 4.87 (CH-1″, d, *J* = 4.9 Hz), δ 5.04 (CH-2′, dd, *J* = 10.5, 7.5 Hz), δ 5.29 (CH-13, dd, *J* = 9.2, 4.3 Hz), δ 7.37 (CH-12′, dd, *J =* 7.7 Hz), δ 7.54 (CH-13′, d, *J =* 7.7 Hz), δ 7.90 (CH-11′, d, *J =* 7.7 Hz), δ 7.94 (CH-15′, s); *m/z* 921 [M + H]^+^; R_f_ 0.61 (EtOAc-CH_3_OH-25% NH_3_, 85:10:5); Anal. Calcd for C_49_H_80_N_2_O_14_ (%): C 64.02, H 8.79, N 4.57. Found (%): C 63.84, H 8.71, N 4.51.

#### 2.3.4. Synthesis of Erythromycin B 2′-[3-(dimethylaminomethyl)benzoate] (EBDMAMB)

A mixture of erythromycin B 2′-(3-chloromethylbenzoate) (2 g), dimethylamine (3.9 mL of a 33% solution in ethanol) (Fluka, Loughborough, UK), and sodium iodide (20 mg) in 100 mL acetone (previously dried over anhydrous Na_2_SO_4_) was refluxed for 20 h. Following the water work-up, the organic layer was reduced to dryness *in vacuo*, and the colourless crystals of EBDMAMB were recrystallised from ethyl acetate. Yield 65%, m.p.128–130 °C, ^1^H NMR (CDCl_3_, 500 MHz): δ 0.71 (CH_3_-17, d, *J* = 7.1 Hz), δ 0.73 (CH_3_-21, d, *J* = 7.5 Hz), δ 0.82 (CH_3_-15, t, *J* = 7.4 Hz), δ 0.96 (CH_3_-20, d, *J* = 6.8 Hz), δ 1.12 (CH_3_-19, d, *J* = 7.1 Hz), δ 1.16 (CH_3_-16, d, *J* = 7.1 Hz), δ 1.26 (CH_3_-6′, d, *J* = 6.2 Hz), δ 1.27 (CH_3_-7″, s), δ 1.30 (CH_3_-6″, d, *J* = 6.2 Hz), δ 1.46 (CH_3_-18, s), δ 2.26 (N(CH_3_)_2_-7′,8′, s), δ 3.05 (CH-4″, d, *J* = 9.2 Hz), δ 3.41 (OCH_3_-8″, s), δ 3.54 (CH-5, d, *J* = 7.5 Hz), δ 3.55 (CH-5′, m), δ 3.69 (CH-11, d, *J* = 9.6 Hz), δ 3.98 (CH-3, dd, *J* = 9.0, 1.5 Hz), δ 4.0 (CH-5″, dq, *J* = 9.0, 6.2 Hz), δ 4.61 (CH-16′, s), δ 4.7 (CH-1′, d, *J* = 7.5 Hz), δ 4.87 (CH-1″, d, *J* = 4.5 Hz), δ 5.04 (CH-2′, dd, *J* = 10.5, 7.5 Hz), δ 5.28 (CH-13, dd, *J* = 9.4, 4.3 Hz), δ 7.39 (CH-12′, dd, *J =* 7.7 Hz), δ 7.54 (CH-13′, bd, *J =* 7.6 Hz), δ 7.91 (CH-11′, d, *J =* 7.6 Hz), δ 7.96 (CH-15′, s); *m/z* 879 [M + H]^+^; R_f_ 0.3 (EtOAc-CH_3_OH-25% NH_3_, 85:10:5); Anal. Calcd for C_47_H_78_N_2_O_13_ (%): C 64.20, H 8.96, N 3.18. Found (%): C 64.49, H 8.98, N 3.19.

#### 2.3.5. Synthesis of 5-Desosaminyl Erythronolide B Ethyl Succinate

5-Desosaminyl erythronolide B ethyl succinate was made from erythromycin B ethyl succinate under similar conditions previously described [17,25,26].

#### 2.3.6. Synthesis of Erythromycin B Enol Ether

Erythromycin B enol ether was made from erythromycin B under conditions previously described [25].

#### 2.3.7. Synthesis of 8-*d*-erythromycin B (8D-EB)

8-*d*-erythromycin B was made from erythromycin B enol ether under conditions previously described [27].

### 2.4. In Vitro Determination of the Anti-Malarial Activity of Selected Erythromycin B Derivatives

#### 2.4.1. Cultivation of *P. falciparum* Parasite

Chloroquine, pyrimethamine and sulfadoxine-resistant K1 strain of *P. falciparum* was cultured with RPMI (Roswell Park Memorial Institute) 1640 media containing 25 mM (2-hydroxyethyl) piperazine-*N*’-(2-ethane-sulfonic acid) (HEPES) and 0.3 g/L L-glutamine (Gibco, Life Technologies, Renfrew, UK). The medium was supplemented with sterile filtered 2.5 g AlbuMax (Sigma-Aldrich, Saint Louis, MO, USA), 2.5 mL hypoxanthine (Sigma-Aldrich, Saint Louis, MO, USA), 2.5 mL 40% glucose (Dextrose Anhydrous, Fisher Scientific, UK) and 0.5 mL gentamycin (Sigma-Aldrich, Saint Louis, MO, USA). Human erythrocytes served as host cells. Cultures were maintained at 37 °C under the gas mixture of 5% CO_2_ and 5% O_2_ in N_2_ gas mixture (BOC Limited, Guildford, UK).

#### 2.4.2. Synchronisation of K1 *P. falciparum*

The K1 strain of *P. falciparum* was cultivated until ≥8% parasitaemia was obtained, with neat prevalence of ring stages. The culture was then transferred to a falcon tube and centrifuged at 3500 rpm for 5 min at 20 °C. Supernatant was removed, and the pellet was re-suspended in 5 mL of aqueous 5% sorbitol at room temperature for 5 min and further centrifuged as before. The supernatant was removed, and the pellet was washed 3 times with complete media before setting up a new culture. The pellet was re-suspended in complete media with enough purified 50% RBCs (red blood cells) to obtain 5% final haematocrit.

#### 2.4.3. Determination of IC_50_ in K1 *P. falciparum*

Here, 1% infected RBCs with *P. falciparum* K1 strain at ring stage were exposed to 9 dilutions at concentrations of 3.05 nM–200 µM (4-fold serial dilution) of compounds from 50 mM stock solutions. 100 µL of culture volume was added in 96-well plates with 100 µL drug dilutions. Untreated infected RBCs served as positive control while the uninfected RBCs was a negative control. The plates were incubated for 72 h at 37 °C, 5% CO_2_, and 5% O_2_ in N_2_. Following the incubation, 150 µL of media was carefully removed, and 150 µL of SYBR (Synergy Brands) Green solution (prepared by adding 2 µL of 10,000 X SYBR Green in 4 mL of phosphate buffer saline (PBS)) was added to each well. The plates were kept in the dark for 45 min at room temperature. The fluorescence intensity was measured using a microplate reader (Genius Tecan) set at 485 nm excitation and 535 nm emission wavelengths. Chloroquine was included as a control drug.

The IC_50_ values were determined using Graph Pad Prism 5.0. Values were calculated using non-linear regression by using *log*-transformed drug concentration plotted against dose-response. Parasitaemia was calculated and normalised relative to response on the controls (cultures without drug).

### 2.5. Conformational Search Studies

#### 2.5.1. Unconstrained Conformational Search

Conformational analysis of selected derivatives of erythromycin B (erythromycin B 9-oxime, 5-desosaminyl erythronolide B ethyl succinate, erythromycin B 2′-[3-(morpholinomethyl)benzoate], erythromycin B 2′-[3-(dimethylaminomethyl)benzoate], and 8-*d*-erythromycin B) was performed using Macromodel under Schrodinger Suite 2021-1 and Maestro v. 12.7.156 as the interface. Chloroform was used as a solvent. The minimisations were first performed with charges from force field, cut off was extended, minimisation method was TNCG (Truncated Newton Conjugate Gradient), and the number of maximum iterations was set to 10,000, with the gradient convergence, and its threshold 0.05. Conformational search torsional sampling was MCMM (Monte Carlo multiple minimum) with automatic setup during the calculation, and torsion sampling options: intermediate. The maximum number of steps was 10,000, with 100 steps per rotatable bond. Number of structures to be saved for each search was 100, energy window for saving structures 21 kJ/mol, and maximum atom deviation cut off 0.5 Å.

#### 2.5.2. Constrained Conformational Search

The constrained conformational search was performed for erythromycin B 2′-[3-(dimethylaminomethyl)benzoate] in chloroform using Macromodel and Maestro v. 12.7.156 as the interface under Schrodinger Suite 2021-1. The distance H4-H11 was constrained to 2.5 ± 0.3 Å and H5-H18 to 2.5 ± 0.3 Å starting from the global minimum of the unconstrained conformational search for this compound.

Conformational search torsional sampling was MCMM with automatic setup during the calculation and torsion sampling options: intermediate. The maximum number of steps was 10,000, with 100 steps per rotatable bond. Number of structures to be saved for each search was 100, energy window for saving structures 21 kJ/mol, and maximum atom deviation cut off 0.5 Å.

### 2.6. Molecular Docking Studies

Molecular docking studies were performed using the Patchdock web server with the Patchdock molecular docking algorithm based on shape complementarity principles [28,29,30]. The input is set consisting of the pdb files of the receptor molecule and the ligand molecule. The pdb files of the ligand molecules consisted of folded-out structures obtained in the unconstrained conformational search for all investigated erythromycin B derivatives, except erythromycin B 2′-[3-(dimethylaminomethyl)benzoate], where we used the global minimum of the constrained conformational search. The output is a list of the potential complexes sorted by the shape complementarity principles.

Ten best results from docking analysis using Patchdock algorithm were submitted to FireDock for refinement [31,32].

Visualisation of the results and the formation of 2D diagrams were performed using Discovery Studio 2021 Client (Dessault Systems Biovia Corp., San Diego, CA, USA).

## 3. Results and Discussion

### 3.1. Synthesis of N-Substituted 2′-(3-aminomethylbenzoate) Esters of Erythromycin B

**Scheme 1:**
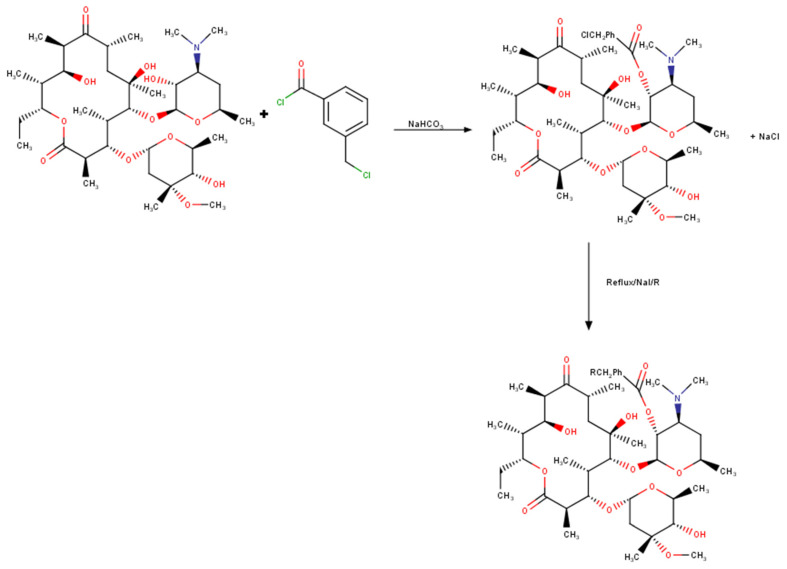
Synthesis of *N*-substituted 2′-(3-aminomethylbenzoate) esters from erythromycin B as a starting macrolide.

*N*-substituted 2′-(3-aminomethylbenzoate) esters of erythromycin B were synthesised in two steps. In the first step, erythromycin B was esterified at C-2′ with 3-chloromethylbenzoyl chloride. In the second step, the product erythromycin B 2′-[3-chloromethylbenzoate] was heated at reflux with the appropriate amine (R) in the presence of sodium iodide as a catalyst (Scheme 1) [33].

### 3.2. In Vitro Investigation of Synthesised Erythromycin B Derivatives against P. falciparum K1 Strain

Erythromycin B and its derivatives were shown to possess anti-bacterial activity [17], but they have never been investigated as anti-malarials. Due to the fact that erythromycin B possesses acid stability [17], we hypothesised that they would show better anti-malarial activity when compared with erythromycin A. Therefore, we selected several derivatives of erythromycin B for evaluation: erythromycin B 9-oxime, 5-desosaminyl erythronolide B ethyl succinate, erythromycin B 2′-[3-(morpholinomethyl)benzoate], erythromycin B 2′-[3-(dimethylaminomethyl)benzoate], and 8-*d*-erythromycin B.

A 1% parasitised culture at ring stage was treated with the compounds for 72 h at concentrations of 3.05 nM–200 μM (four-fold serial dilution) against the *P. falciparum* K1 strain, and the growth was measured using the SYBR Green-based plate reader assay.

SYBR Green is a sensitive DNA stain that can bind to the double-stranded parasite DNA, and once intercalated, it becomes fluorescent [34]. SYBR Green assay offers a quick, inexpensive, and straightforward method to test parasites’ susceptibility of anti-malarial drugs [35]. The assay takes the advantage of the fact that the parasite–host cells (healthy human mature erythrocytes) lack DNA; therefore, the fluorescence intensity reflects parasite growth.

**Figure 2:**
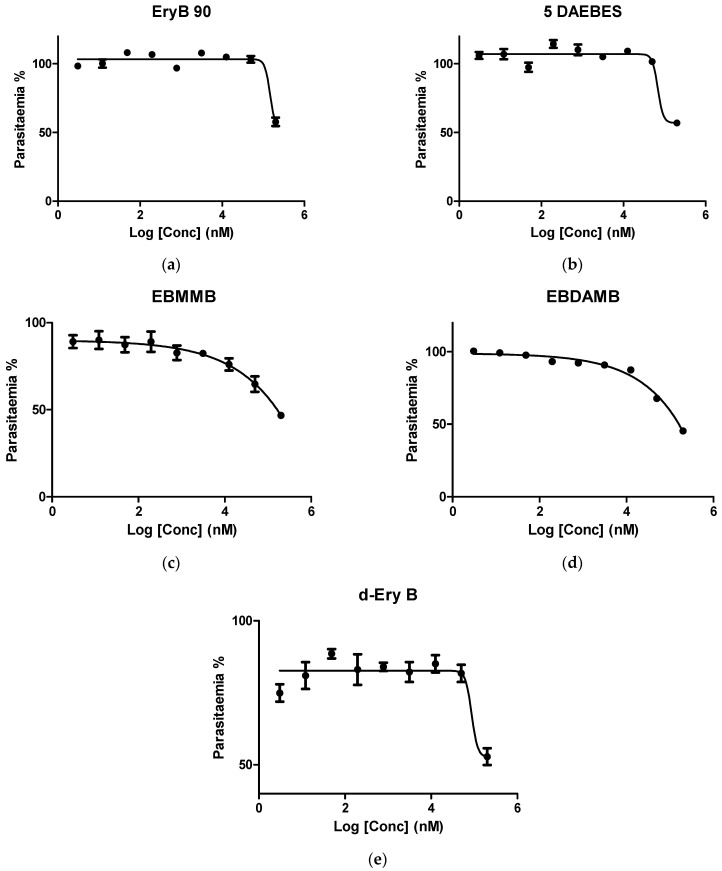
Dose-response curves for (**a**) erythromycin B 9-oxime (EryB 90), (**b**) 5-desosaminyl erythronolide B ethyl succinate (5DAEBES), (**c**) erythromycin B 2′-[3-(morpholinomethyl) benzoate] (EBMMB), (**d**) erythromycin B 2′-[3-(dimethylaminomethyl)benzoate] (EBDAMB), and (**e**) 8-*d*-erythromycin B (d-Ery B).

The investigated compounds exhibited anti-malarial activities with micromolar IC_50_ values against the multi-drug-resistant *P. falciparum* strain K1 (Figure 2). Erythromycin B 9-oxime gave an IC_50_ value of 146.0 μM; 5-desosaminyl erythronolide B ethyl succinate gave an IC_50_ value of 68.6 μM; and 8-*d*-erythromycin B gave an IC_50_ of 86.8 μM. Both erythromycin B 2′-[3-(morpholinomethyl)benzoate] and erythromycin B 2′-[3-(dimethylaminomethyl)benzoate] were less active, with IC_50_ values of more than 200 μM.

### 3.3. Conformational Search Studies of the Investigated Derivatives of Erythromycin B in Chloroform

#### 3.3.1. Unconstrained Conformational Search of the Investigated Derivatives of Erythromycin B in Chloroform

We chose chloroform as a solvent because it was previously found that the most commonly used dielectric constant for proteins is 4; inside the protein according to the recent investigation, it is 6–7 [36]; and inside nucleic acids, it is 2 [37].

Folded-out and folded-in terms were defined by Everett et al. (1990) [38], and it was shown that erythromycin A mainly possesses characteristics of the folded-out conformational type [39]. The distances (Å) among the characteristic hydrogens for folded-out and folded-in conformations were measured for selected derivatives of erythromycin B in chloroform as a solvent, which are given in Table 1, together with the energies of global minima and the number of times they repeat.

The global minimum of erythromycin 9-oxime in chloroform is neither folded out nor folded in, although the H4-H18 and H3-H8 distances correspond to a typical folded-in conformation. Erythromycin B 2′-[3-(morpholinomethyl) benzoate] in chloroform has a global minimum, which is a variation of a folded-out conformation. The global minimum of erythromycin B 2′-[3-(dimethylaminomethyl)benzoate] in chloroform is a typical folded-in conformation. 5-Desosaminyl erythronolide B ethyl succinate in chloroform shows a global minimum that is neither folded-out nor folded-in, but is different from the global minimum of erythromycin B 2′-[3-(morpholinomethyl) benzoate]. The global minimum of 8-*d*-erythromycin B is a folded-in conformation.

Previously, it was shown that the active conformation of the macrolide antibiotics bound weakly to bacterial ribosomes, such as clarithromycin, erythromycin A, and azithromycin [39], is mainly the folded-out conformation. None of the global minima for the investigated derivatives of erythromycin B is a typical folded-out conformation. Typical folded-out conformations for erythromycin B and 8-*d*-erythromycin B were found bound weakly to the bacterial ribosomes [27]; therefore, folded-out conformations were used for the docking analysis as the active conformations of the ligands-investigated derivatives of erythromycin B (erythromycin B 9-oxime, erythromycin B 2′-[3-(morpholinomethyl) benzoate], erythromycin B 2′-[3-(dimethylaminomethyl)benzoate], 5-desosaminyl erythronolide B ethyl succinate, and 8-*d*-erythromycin B) to the complex consisting of 23S rRNA and L4 protein from the apicoplast ribosome of *P. falciparum* previously constructed *in silico* [40]. The typical folded-out conformation (H4-H11 (2.37 Å), H5-H18 (2.58 Å), H15-H16 (2.92 Å)) of erythromycin B 9-oxime was found with the energy 128.73 kJ/mol and erythromycin B 2′-[3-(morpholinomethyl) benzoate] with the energy 103.57 kJ/mol (H4-H11 (2.77 Å), H5-H18 (2.54 Å), H15-H16 (3.05 Å)). Among the first 50 conformations of the unconstrained conformational search of erythromycin B 2′-[3-(dimethylaminomethyl)benzoate], the typical folded-out conformation was not found—the most similar was found with the energy 71.84 kJ/mol (H4-H11 (2.60 Å), H5-H18 (3.47 Å), and H15-H16 (4.54 Å)). 5-Desosaminyl erythronolide B ethyl succinate shows the typical folded-out conformation with the energy 110.47 kJ/mol (H4-H11 (2.24 Å), H5-H18 (2.64 Å), H15-H16 (3.06 Å)), and 8-*d*-erythromycin B with the energy 112.5 kJ/mol (H4-H11 (2.82 Å), H5-H18 (2.61 Å), and H15-H16 (3.13 Å)).

#### 3.3.2. Constrained Conformational Search of Erythromycin B 2′-[3-(dimethylaminomethyl)benzoate]

The constrained conformational search on erythromycin B 2′-[3-(dimethylaminomethyl)benzoate] in chloroform gave a global minimum with the energy 69.02 kJ/mol repeated two times. It is a folded-out structure (H4-H11 (2.79 Å), H5-H18 (2.54 Å), and H15-H16 (3.04 Å)).

### 3.4. Docking Analysis of the Investigated Derivatives of Erythromycin B into In Silico Constructed Segment of the Exit Tunnel from the Apicoplast Ribosome of P. falciparum

The docking analysis of erythromycin B derivatives into *in silico* constructed exit tunnel from apicoplast ribosome from *P. falciparum* was complex. The majority of free or commercially available software for molecular docking are either suitable for proteins or RNAs as receptors, but not for complexes which contain both protein and RNA residues. We chose PatchDock for our docking analysis because it relies on different principles—on shape complementarity [26,27,28]—compared to other software.

For all docking analyses with derivatives of erythromycin B as ligands and complexes containing protein and three domains of 23S rRNAs as receptors, results were characterised with the parameters presented in Table 2, such as score, area, and ACE.

Ten best results of performed molecular docking for each investigated erythromycin B derivatives were subjected to FireDock refinement. Solution number, global energy, attractive van der Waals, repulsive van der Waals, and ACE for investigated erythromycin B derivatives are given in Table 3.

**Figure 3:**
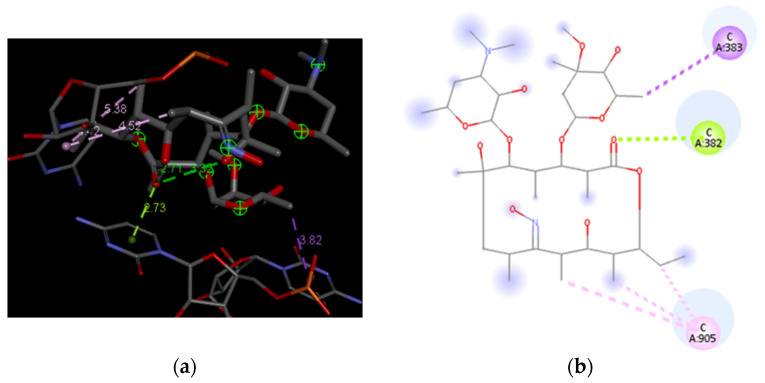
(**a**) Graphical representation of the obtained interactions between erythromycin B 9-oxime and the segment of the exit tunnel from apicoplast ribosome of *P. falciparum* with distances in Å; (**b**) 2D diagram of receptor–ligand interactions.

**Figure 4:**
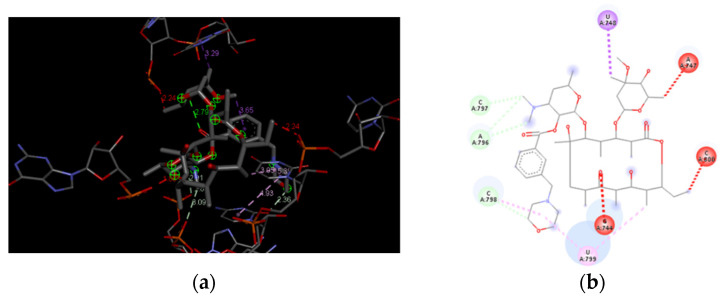
(**a**) Graphical representation of the obtained interactions between erythromycin B 2′-[3-(morpholinomethyl)benzoate] and the segment of the exit tunnel from apicoplast ribosome of *P. falciparum* with distances in Å; (**b**) 2D diagram of receptor–ligand interactions.

Erythromycin B 9-oxime shows in the best-ranked solution after Firedock refinement without predetermined binding sites only interactions with nucleotides: π-σ (violet circles), and π-Lone pair (light green circles) and π-alkyl (pink circles) (Figure 3). Erythromycin B 2′-[3-(morpholinomethyl)benzoate] under the same circumstances shows the solution with the following interactions with nucleotides: π-σ (violet circles), π-alkyl (pink circles), and carbon-hydrogen bond (light blue circles), as well as unfavourable bumps (red circles) (Figure 4).

**Figure 5:**
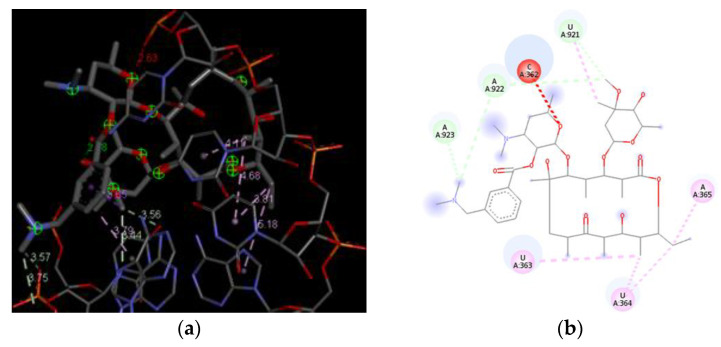
(**a**) Graphical representation of the obtained interactions between erythromycin B 2′-[3-(dimethylaminomethyl)benzoate] and the segment of the exit tunnel from apicoplast ribosome of *P. falciparum* with distances in Å; (**b**) 2D diagram of receptor–ligand interactions.

**Figure 6:**
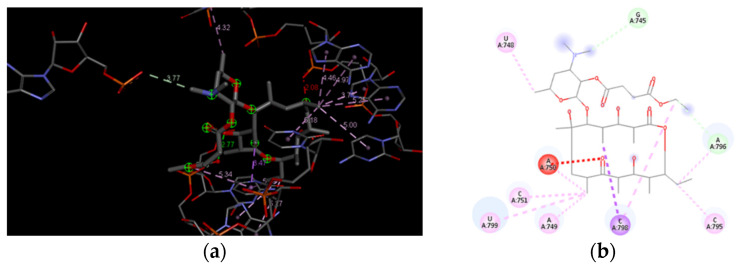
(**a**) Graphical representation of the obtained interactions between 5-desosaminyl erythronolide B ethyl succinate and the segment of the exit tunnel from apicoplast ribosome of *P. falciparum* with distances in Å; (**b**) 2D diagram of receptor–ligand interactions.

Erythromycin B 2′-[3-(dimethylaminomethyl)benzoate] shows very similar behaviour regarding present interactions in the best-ranked solution structure after refinement using Firedock algorithm with the receptor: π-alkyl (pink circles), carbon hydrogen bond (light-blue circles), and an unfavourable bump (red circle) (Figure 5). 5-Desosaminyl erythronolide B ethyl succinate shows the following interactions with the nucleotides in the best-ranked solution: π-σ (violet circles), π-alkyl (pink circles), and carbon hydrogen bond (light-blue circles), as well as an unfavourable bump (red circle) (Figure 6).

**Figure 7:**
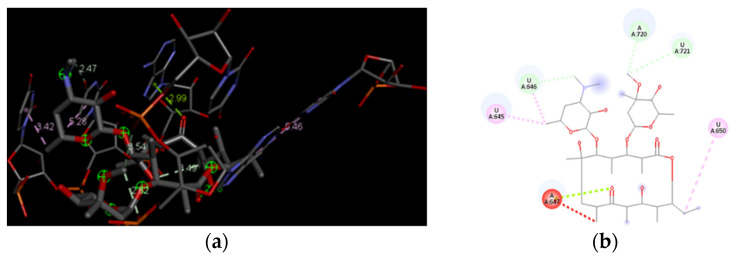
(**a**) Graphical representation of the obtained interactions between 8-*d*-erythromycin B and the segment of the exit tunnel from apicoplast ribosome of *P. falciparum* with distances in Å; (**b**) 2D diagram of receptor–ligand interactions.

8-*d*-Erythromycin B shows some general patterns regarding the interactions with the nucleotides from the receptor—*in silico* made segment of the exit tunnel from apicoplast ribosome of *P. falciparum*: unfavourable bump (red circle), carbon hydrogen bond (light blue circle), π-Lone pair (light green circle), and π-alkyl (pink circle) (Figure 7).

The docking results, followed by the refinement, provide a good explanation for the results of the anti-malarial tests. The highest number of unfavourable interactions is shown by erythromycin B 2′-[3-(morpholinomethyl)benzoate]; therefore, it is not surprising that this compound does not show any anti-malarial activity (IC_50_ > 200 μM). Erythromycin B 9-oxime does not show any unfavourable interactions, different from others, but it shows only five favourable interactions, the lowest number among the investigated erythromycin B derivatives. The highest number of favourable interactions was shown in case of 5-desosaminyl erythronolide B ethyl succinate; therefore, it is in good correlation with found IC_50_ value, which is the lowest among the investigated derivatives, i.e., the highest anti-malarial activity among the tested compounds. Erythromycin B 2′-[3-(dimethylaminomethyl)benzoate] shows a higher number of favourable interactions (8) compared to 8-*d*-erythromycin B (7), but the last derivative shows interaction with more contribution (π-Lone pair); therefore, this is the reason why 8-*d*-erythromycin B shows better anti-malarial activity compared to erythromycin B 2′-[3-(dimethylaminomethyl)benzoate]. π-Lone pair is the interaction shared both between 8-*d*-erythromycin B and erythromycin B 9-oxime, but because of the higher number of favourable interactions shown by 8-*d*-erythromycin B, it shows better anti-malarial activity.

It can be speculated that the small number of favourable interactions or the presence of unfavourable interactions is the reason for weak anti-malarial activity of investigated derivatives of erythromycin B. Molecular docking can also be a good tool for the prediction of anti-malarial activity of derivatives with previously undetermined anti-malarial activity. In the future, we will broaden our investigation using both *in silico* tools and experimental results.

## 4. Conclusions

Five derivatives of erythromycin B were synthesised and evaluated for their anti-malarial activity against multi-drug-resistant K1 strain of *P. falciparum*. They do not show significant anti-malarial activity, ranging from 68.6 μM for 5-desosaminyl erythronolide B ethyl succinate to more than 200 μM for erythromycin B 2′-[3-(morpholinomethyl)benzoate] and erythromycin B 2′-[3-(dimethylaminomethyl)benzoate]. Performed conformational search (unconstrained for all and constrained only for erythromycin B 2′-[3-(dimethylaminomethyl)benzoate]) in chloroform gave the most probable folded-out structures which were used as active conformations for molecular docking. Molecular docking results provided an explanation for the order of anti-malarial activity amongst the investigated derivatives of erythromycin B.

## Figures and Tables

**Table 1:** Distances (Å) for the selected contacts of the global minima obtained in the unconstrained conformational search for selected derivatives of erythromycin B, and energies of global minima and times they repeat.

Compounds	Global Minimum		Distances (Å)						
	Energy (kJ/mol)	Number of Repeats	H4-H11	H15-H16	H3-H8	H3-H11	H4-H18	H5-H18	H16-H17
Erythromycin B 9-oxime	124.27	3	6.70	4.59	2.20	5.18	2.77	3.46	2.93
Erythromycin B 2′-[3-(morpholinomethyl) benzoate]	102.62	2	3.02	2.88	5.67	3.92	4.08	2.57	3.83
Erythromycin B 2′-[3-(dimethylaminomethyl) benzoate]	70.16	4	4.79	3.69	2.34	2.15	2.51	3.48	2.57
5-Desosaminyl erythronolide B ethyl succinate	−118.56	1	3.82	3.05	6.93	4.72	3.55	3.44	4.99
8-*d*-Erythromycin B	111.13	1	3.97	3.76	2.35 *	2.19	2.73	3.47	2.26

* H3-D8.

**Table 2:** Score, area, and ACE (atomic contact energy) of top ten results based on shape complementarity principles for docking analysis of investigated derivatives of erythromycin B with complex of protein and three domains of 23S rRNA.

Solutions	Solution Parameters	Derivatives				
		EryB 90	EBMMB	EBDAMB	5DAEBES	d-Ery B
1	Score	7332	7578	7362	6430	7132
	Area	886.20	972.00	1012.70	740.40	800.00
	ACE	−341.11	−477.95	−490.63	−324.01	−337.27
2	Score	7032	7388	7030	6222	6416
	Area	839.20	951.20	745.90	739.90	754.20
	ACE	−339.59	−419.55	−275.08	−467.41	−362.32
3	Score	6640	7376	6980	6162	6296
	Area	845.50	896.30	785.80	865.40	900.70
	ACE	−277.37	−424.72	−376.62	−404.81	−342.87
4	Score	6636	7276	6970	6110	6272
	Area	700.10	966.70	827.10	840.20	749.90
	ACE	−326.43	−462.36	−437.15	−442.39	−264.42
5	Score	6598	6834	6954	6056	6260
	Area	896.20	898.30	860.40	727.70	836.30
	ACE	−408.91	−461.77	−418.69	−365.69	−280.40
6	Score	6556	6830	6904	6000	6254
	Area	704.80	814.00	858.30	759.10	701.70
	ACE	−379.30	−311.01	−431.74	−286.73	−325.53
7	Score	6554	6826	6896	5972	6250
	Area	719.30	891.70	816.60	831.00	736.90
	ACE	−336.11	−334.69	−362.42	−319.28	−386.86
8	Score	6524	6824	6870	5952	6212
	Area	802.30	885.40	812.00	716.10	722.80
	ACE	−464.79	−330.79	−351.92	−351.59	−353.71
9	Score	6500	6726	6868	5896	6190
	Area	786.90	754.70	851.10	646.80	670.10
	ACE	−389.76	−291.68	−349.78	−267.87	−306.69
10	Score	6462	6722	6790	5892	6122
	Area	714.60	1011.00	1014.50	693.60	804.90
	ACE	−372.85	−489.58	−487.56	−183.95	−351.11

**Table 3:** Solution number, global energy, attractive Van der Waals, repulsive van der Waals, and ACE for investigated erythromycin B derivatives with *in silico* constructed segment of the exit tunnel from the apicoplast ribosome of *P. falciparum*.

Rank	Derivatives																								
	eryB9O					eryBMB					eryBDA					eryBD					eryB8D				
	SN	GE	AVdW	RVdW	ACE	SN	GE	AVdW	RVdW	ACE	SN	GE	AVdW	RVdW	ACE	SN	GE	AVdW	RVdW	ACE	SN	GE	AVdW	RVdW	ACE
1	10	−63.18	−23.29	9.23	−22.58	5	−74.06	−28.32	8.97	−24.41	5	−57.60	−23.55	13.89	−21.13	2	−67.89	−25.26	11.31	−24.74	6	−63.40	−26.31	9.46	−20.67
2	9	−60.65	−24.69	9.32	−20.27	3	−55.61	−21.65	7.65	−19.85	4	−52.66	−20.91	14.62	−20.77	1	−52.42	−20.56	9.31	−18.29	1	−61.51	−23.48	8.48	−20.95
3	1	−60.28	−24.59	8.05	−19.29	4	−50.21	−26.49	26.04	−19.84	8	−51.82	−21.02	4.11	−15.61	8	−49.83	−22.88	22.20	−20.92	2	−48.35	−21.82	17.42	−18.82
4	2	−59.82	−23.60	12.36	−21.84	9	−44.94	−18.35	7.22	−15.85	10	−51.58	−28.90	26.43	−20.68	7	−47.89	−23.46	14.09	−16.53	8	−46.70	−20.56	18.10	−19.11
5	8	−53.61	−23.03	16.31	−21.13	7	−44.11	−24.22	27.44	−18.84	6	−48.66	−23.05	16.02	−19.41	3	−45.82	−19.87	8.91	−15.22	9	−45.24	−15.63	3.87	−15.69
6	6	−47.33	−16.88	9.54	−18.68	6	−41.75	−23.33	15.91	−14.88	2	−48.50	−18.58	4.20	−15.64	10	−44.62	−22.38	8.79	−14.04	7	−42.49	−17.24	11.94	−16.47
7	4	−44.88	−16.35	7.59	−16.79	2	−30.70	−25.80	40.54	−17.23	3	−45.67	−22.70	23.01	−19.13	9	−38.29	−17.57	11.63	−14.30	4	−40.76	−19.02	11.57	−15.62
8	7	−42.39	−16.63	9.09	−15.56	10	−29.42	−18.97	19.66	−12.45	7	−41.62	−19.57	13.90	−16.31	5	−38.09	−21.25	25.36	−18.31	3	−33.22	−14.06	6.78	−13.20
9	3	−23.15	−7.92	2.81	−9.12	8	−21.96	−11.50	4.54	−5.58	1	−40.86	−25.65	23.11	−15.79	4	−34.84	−14.83	7.62	−12.25	5	−24.99	−12.77	12.30	−11.45
10	5	−15.07	−9.36	7.03	−4.80	1	−15.89	−27.10	72.48	−21.55	9	−24.27	−8.63	3.70	−9.54	6	−32.26	−20.80	22.81	−13.81	10	−11.66	−22.26	59.71	−16.76

Abbreviations used: eryB9O: erythromycin B 9-oxime, eryBD: 5-desosaminyl erythronolide B ethyl succinate, eryBMB: erythromycin B 2′-[3-(morpholinomethyl)benzoate], eryBDA: erythromycin B 2′-[3-(dimethylaminomethyl)benzoate], eryB8D: 8-*d*-erythromycin B, SN: solution number, GE: global energy, AVdW: attractive Van der Waals, RVdW: repulsive Van der Waals, and ACE: atomic contact energy.

## Data Availability

All data necessary for understanding of the research are present in the manuscript. Additional data related to molecular modelling and NMR analyses of synthesised compounds can be obtained from authors upon the request.
